# The Impact of the Receptor of Hyaluronan-Mediated Motility (RHAMM) on Human Urothelial Transitional Cell Cancer of the Bladder

**DOI:** 10.1371/journal.pone.0075681

**Published:** 2013-09-17

**Authors:** Christian Niedworok, Inga Kretschmer, Katharina Röck, Frank vom Dorp, Tibor Szarvas, Jochen Heß, Till Freudenberger, Ariane Melchior-Becker, Herbert Rübben, Jens W. Fischer

**Affiliations:** 1 Department of Urology, Essen Medical School, University Duisburg-Essen, Essen, Germany; 2 Institut für Pharmakologie und Klinische Pharmakologie, Universitätsklinikum Düsseldorf, Heinrich-Heine-Universität, Düsseldorf, Germany; 3 Department of Urology, Medical University Vienna, Vienna General Hospital, Vienna, Austria; University of Patras, Greece

## Abstract

Hyaluronan (HA) is a carbohydrate of the extracellular matrix with tumor promoting effects in a variety of cancers. The present study addressed the role of HA matrix for progression and prognosis of human bladder cancer by studying the expression and function of HA-related genes.

**Methods:**

Tissue samples of 120 patients with different stages of transitional cell bladder cancer, who underwent surgical treatment for bladder cancer at the University Hospital of Essen were analysed. mRNA-expression levels of HA synthases (HAS1-3) and HA-receptors (RHAMM and CD44) were evaluated by real time RT-PCR in comparison to healthy bladder tissue as control. In uni- and multivariate cox proportional hazard survival regression analysis, the impact of the gene expression levels on survival was assessed. *In* *vitro* knock-down of RHAMM, CD44 and HAS isoenzymes was achieved by siRNA and lentiviral shRNA in J82 bladder cancer cells. Transfected cells were analysed *in* *vitro* with regard to proliferation, cell cycle and apoptosis. J82 cells after knock-down of RHAMM were xenografted into male nu/nu athymic mice to monitor tumor progression *in* *vivo*.

**Results:**

In invasive tumor stages RHAMM-, HAS1 and HAS2 mRNA-expression levels were elevated whereas HAS3v1 was reduced as compared to non-invasive tumors. Subsequently, Kaplan-Meier analysis revealed reduced bladder cancer specific survival in patients with high RHAMM mRNA and low HAS3v1 expression. Elevated RHAMM in invasive tumors was confirmed by RHAMM immunohistochemistry. Furthermore, multivariate analysis revealed that only RHAMM expression was associated with poor prognosis independent from other survival factors (HR=2.389, 95% CI 1.227-4.651, p=0.01). Lentiviral RHAMM knock-down revealed reduced J82 cell proliferation *in* *vitro* and reduced xenograft tumor growth *in* *vivo*.

**Conclusion:**

The data suggest that RHAMM plays a crucial role in mediating progression of muscle-invasive bladder cancer and recommends RHAMM for further evaluation as a prognostic marker or therapeutic target in bladder cancer therapy.

## Introduction

Transitional cell carcinoma (TCC) of the bladder is the ninth most common malignant disease with a global 5-year prevalence of 860,299 males and 249,966 females [1]. In non-muscle-invasive tumors (Ta) progression to muscle-invasive disease (T2-T4) is rare and occurs in 3 to 5% of all cases [2]. While patients with genetically stable nonmuscle invasive low grade tumors show an excellent 5 year survival rate of 96%, patients with deep muscle infiltrating high grade tumors have the worst prognosis with a 5 year survival rate of about 20% [2,3]. In exophytic, nonmuscle invasive low grade tumors transurethral resection (TUR) is the local, bladder preserving, therapy of choice. In muscle infiltrating tumors radical cystectomy with extended pelvic lymph node dissection is the generally accepted treatment with curative intent. In patients with severe comorbidities alternative treatment option is the combined chemo- and radiation therapy. Nevertheless muscle infiltrating transitional cell carcinomas of the bladder represent a heterogenous group of tumors with regard to clinical outcome. In patients with pT3/4 tumors about 50% are at risk of developing metastases despite of extended surgery. Therefore, it is of great importance to elucidate the pathophysiology of BC and to develop more precise diagnostic markers for progression of this subset of muscle-invasive carcinomas that confer high risk of cancer specific mortality.

Hyaluronan (HA) is a polymer of alternating N-acetylglucosamine and glucuronic acid residues and is one of the main carbohydrate components of the extracellular matrix (ECM). HA is synthesized by three HA-synthase (HAS) isoenzymes (HAS1, HAS2, HAS3) and is either retained near the cell surface where it forms a pericellular HA-rich microenvironment or is released from the cell surface and deposited in the extracellular matrix. HA itself is not transforming but has been shown to support many important facets of the malignant cell phenotype, such as proliferation, migration and resistance to apoptosis [4]. Even inflammation can be promoted by HA forming supramolecular structures, HA-cables, which bind monocytes and lymphocytes and are therefore thought to enhance inflammation [5].

Different cancers are associated with increased tumor cell or stroma cell associated HA and with differential expression of HAS-isoenzymes [6]. So far it is not clear whether the specific association of HAS isoenzymes with specific cancer entities reflects different biological roles of the HAS isoenzymes or is the result of (i) the presence of different autocrine and paracrine factors and/or (ii) the specific cell types involved in each cancer entity. It is, however, likely that HAS isoenzymes differ with regard to the size of the secreted HA-polymer which could consecutively evoke different biological functions [7]. The most efficient mechanism to modify the length of HA polymers are the hyaluronidases (Hyal) that have been shown to strongly support tumor progression by, generating HA fragments (sHA) that are activators of HA signaling through either CD44 or toll-like receptors. Furthermore, sHA is implicated in tumor angiogenesis which contributes to the tumor supporting effect of HA [8,9]. In general, HA induces cellular signaling through HA receptors such as CD44 and the receptor of hyaluronan-mediated motility (RHAMM). Both HA receptors have been implicated in the progression of cancer likely by promoting malignant cancer cell phenotypes [4,10]. Especially, activation of the ERK1/2 signaling pathway and the PI3K pathway could contribute to the tumor promoting effects of both receptors. RHAMM was identified as receptor involved in cell motility during physiological and malignant processes [11,12]. RHAMM can be associated with the cell surface or function intracellular. RHAMM is involved in ECM induced cell signaling through regulating the stability of focal adhesion complexes [13,14] and activates Ras-, src-, Erk-kinase and protein-kinase-C [15]. In addition, the intracellular form of RHAMM binds to mitotic spindles [16] and regulates mitosis [17]. In this regard both, overexpression and loss of RHAMM, cause perturbation of the mitotic spindle and subsequently genetic instability [17]. Whereas HA is the principle ligand of RHAMM, CD44 binds also various other ligands such as osteopontin, fibronectin or collagen [18]. Interestingly, in some cases RHAMM and CD44 appear even to cooperate with respect to signaling [19]. In addition, HA has been attributed a role in mediating chemoresistance either by controlling the diffusion of anti-cancer drugs [20] and/or by affecting multi-drug resistance transporters that mediate efflux of xenobiotics [20,21].

BC has been studied earlier with respect to HA and HA-associated genes. Previous studies revealed that especially the HAS1 isoenzyme is associated with BC progression [22,23]. Furthermore, urinary excretion of HA was established as an indicator of poor prognosis in BC [24]. Recently, again HAS1 mRNA expression was associated with BC metastasis [25]. Another important finding is that Hyal1 has a detrimental role in human BC and that Hyal1 is associated with high grade BC [26-28]. Moreover, high expression of Hyal1 has recently been suggested to predict progressive muscle-invasive and recurrent BC and to associate with metastasis and decreased disease specific survival [29]. All together, the available data on HA in BC point to increased synthesis of HA mainly by HAS1 and degradation to tumor promoting sHA by Hyal 1. In turn, the effects of HA and sHA are likely transduced through activation of HA-receptors. In BC certain splice variants of CD44, such as CD44V8-10, were upregulated and associated with poor prognosis [30,31]. In addition, it has been shown that CD44 might be involved in the angiogenic response to HA in BC [22]. However, a recent analysis revealed that CD44v was upregulated and CD44s was downregulated without diagnostic or prognostic value [30]. RHAMM has been described to be upregulated but also without clear correlation with clinical parameters [25,32]. Because it appears plausible that the amount of HA-receptors plays an important role in the transduction of HA signaling and thereby in the progression of human BC, the aim of the present study was to study the expression of HA-receptors and HAS isoenzymes and to achieve mechanistic information in a patient collective characterized by >50% muscle-invasive BC.

## Materials and Methods

### Tumor Samples

Gene expression analysis was performed in tissue samples of 120 patients with different stages of transitional cell BC (31xTa, 24xT1, 21xT2, 25xT3, 19xT4), who underwent surgical treatment for BC at the University Clinics of Essen between 1990 and 2004. All frozen tissue samples were cut and stained with H&E to verify their tumor content. Only specimens containing ≥70% tumor cells were subjected to further analysis. In addition, 20 cases of paraffin embedded bladder carcinomas (four of each stage, pTa-pT4) were assessed using immunohistochemistry. The study protocol with the project number 07-3537 was approved by the Ethics Committee of the University of DuisburgEssen, Germany. Each patient provided a written informed consent for storing fresh frozen and paraffined tumor material and the relevant clinical data.

The primary endpoint of this study was cancer-speciﬁc death. Cause of death was obtained from death certiﬁcates. Subjects were followed up from baseline (date of surgery) survey until July 2009. Normal bladder tissue was taken from patients suffering from non-malignant disease (pyelouretral stricture, neurogenic bladder dysfunction).

### Immunohistochemistry

Immunohistochemistry was performed on paraffin embedded BC specimens. For RHAMM staining antigen retrieval in citrate buffer (pH 6.2, containing 0.05% Tween20®) was performed by boiling the slides at 95°C for 25 minutes. Tissue sections were incubated over night at 4°C with primary antibodies, diluted in 2% BSA in PBS (rabbit anti-RHAMM IgG, 1:100, Amsbio®, Lake Forest, USA); rabbit anti-Ki67 IgG, 1:25, (Novus Biologicals, Ltd., Cambridge, UK). After incubation with the primary antibody slides were rinsed, blocked in 3% H_2_O_2_ solution for 5 minutes and incubated with the secondary antibody (conjugated with horseradish peroxidase) for 1 hour at room temperature and rinsed again three times for 5 minutes. For each staining negative control sections with omission of the primary antibody were included. Detection was performed using 3,3'-diaminobenzidin (DAB). At least three representative areas were photographed using a Leica® DM2000 system and quantitative analysis of staining intensity was performed using ImageJ image processing software (National Institutes of Health, Bethesda, Maryland, USA) as previously described [33].

### Real-time-RT-PCR

mRNA was isolated from flash-frozen tumor tissue using RNeasy total RNA kits (Qiagen, Hilden, Germany). RNA concentration was evaluated by photometric measurement at 260/280nm. 1 µg RNA was used for cDNA synthesis using the QuantiTect Reverse Transcription Kit (Qiagen, Hilden, Germany). PCR reactions were performed by using the Platinum® SYBR® Green qPCR SuperMix-UDG (Invitrogen, Karlsruhe, Germany) in the 7300 real-time PCR system (Applied Biosystems, Darmstadt, Germany). Primer sequences of the genes of interest were used as described before [14]. Expression levels were normalized to the GAPDH expression and calculated using the ΔΔC^)^ method.

### Cell culture and knock-down of HA related genes

J82 BC cells are characterized as low grade cells of high malignancy [34] and were obtained from ATCC^®^ (Manassas, VA, USA) and cultured under routine conditions in RPMI medium containing 10% fetal bovine serum. To knock-down RHAMM in human J82 BC cells, short hairpin RNA (shRNA) sequence targeting RHAMM (5′-CCGGCGTCTCCTCTATGAAGAACTACTCGAGTAGTTCTTCATAGAGGAGACGTTTTTG-3′) was cloned into the pLKO.1 vector using the Mission^TM^ lentiviral shRNA knock-down system (Sigma-Aldrich^®^). Scrambled shRNA was used as control. Production of vector, culture of human embryonic kidney cells (HEK-293T), harvest of recombinant particles and lentiviral transduction of human J82 urothelial cancer cells was performed as described before for knock-down of RHAMM [14]. Seven days after lentiviral transduction of J82 cells with shRHAMM the mRNA expression of RHAMM was reduced to less than 40% compared to J82 cells transduced with scrambled controls. Subsequently the cells were used for xenograft experiments. Proliferation was subsequently determined as described before [14]. In additional *in vitro* experiments J82 cells were treated with siRNA to knock-down RHAMM, CD44, HAS1, HAS2 and HAS3. For these experiments the following siRNA sequences (Qiagen, Germany) were used: siRHAMM (Hs_HMMR_9); siHAS1 (Hs_HAS1_4); siHAS2 (Hs_HAS2_4); siHAS3 (Hs-HAS3_7); siCD44 (Hs_CD44_5); control (Ctrl_AllStars_1).

To inhibit HA synthesis in J82 cells 4-methylumbelliferone (4-MU) dissolved in DMSO was applied (300 µM). Furthermore, in selected experiments exogenous HA (Healon 5, Abbott) was used at a concentration of 100 µg/mL.

### Cell cycle analysis and apoptosis

Cell cycle progression was analysed after knock-down of RHAMM and expressed as percentage of cells in G0/G1 and G2 phase. Briefly nuclei of J82 cells were labeled with Guava Cell Cycle Reagent (Millipore) according to the manufacturer’s protocol and samples were analyzed using a Guava easyCyte Flow Cytometer. Apoptosis was assessed by percentage of cells in sub G1 phase [35]. For PARP immunoblotting polyclonal PARP antibody (1:1000, Cell Signaling, #9542) was used as a primary antibody and detection was visualised by goat anti-rabbit secondary antibody conjugated to IR-Dye 800 (1:10000, LI-COR Biosciences) with the use of Odyssey Near Infrared Imaging System (LI-COR Biosciences).

### Xenograft animal model

Male nu/nu athymic mice (8-12 weeks old) were used for xenografting of J82 cells. 100 µl PBS con-taining 1x10^6^ tumor cells were applied subcutaneously into both flanks of the animals. Tumor growth was monitored for 45 weeks. During this time animals were monitored for excessive tumor growth (> 15 mm diameter), ulcerations, marked weight loss (>20%) or clinical signs of pain and discomfort, which would have been reasons to sacrifice affected individuals. Six animals per group (knock-down and control) received the cells 7 days after lentiviral knock-down as described above. Tumor growth was measured with a caliper twice a week for 45 days. Subsequently, the animals were sacrificed (cervical dislocation), tumors were harvested and fixed for immunohistological staining. Samples of liver and lung were obtained to search for metastatic tumor growth by staining. The mice experiments were performed in accordance with the rules and standards of the LANUV (Landesamt für Natur, Umwelt und Verbraucherschutz, NRW) and the Heinrich-Heine-Universität Düsseldorf. The LANUV approved the experiments and confirmed the compliance of quality standards. The project is identified with the number 87-51.04.2010.A149.

### Statistical analysis

Gene expression data were analyzed either by analysis of variance and the Bonferroni post-hoc test or by Student’s *t* test as appropriate. Data are presented as means ± SEM. Statistical significance was assigned at the level of *p* < 0.05. Data concerning clinical results from patient parameters were lacking normal distribution and were therefore analysed by the non-parametric two-tailed Wilkoxon rank sum test (Mann-Whitney) for paired group comparisons. Stage-dependent correlations were calculated using Pearson’s correlation testing. Analysis of overall survival and disease-specific survival was performed using both, epidemiologic uni- and multivariate Cox proportional hazard survival regression analysis and Kaplan-Meier survival probability test, IBM® SPSS® statistical analysis software (version 18.0, Chicago, IL, USA).

## Results

### Patient Characteristics

A total of 55 patients suffered from superficial disease (pTa and pT1), 65 patients were diagnosed with muscle-invasive disease (unequally distributed in 21 patients with pT2-disease, 25 pT3 and 19 pT4). Tumors were of low grade (G1/G2) in 63 or high grade (G3) in 57 cases. Lymph node positivity was present in 23/120 patients. 74 patients suffered from primary BC while 46 patients experienced a recurrent disease. 55 patients were treated by transurethral resection of the TCC, in 65 cases radical cystectomy was performed. Smoking status was known in 83 patients, 49 were smokers and 34 were non-smokers. Median follow-up time was 34 months, maximum time of follow-up was 189 months. Median patient’s age was 65 years (32-87 years). The clinical data of patient characteristics are shown as part of Table 1.

**Table 1:** RHAMM mRNA expression levels are elevated in muscle-invasive tumor stages, high-grade tumors, positive lymph node status and tumor recurrence.

	**n**	**RHAMM**	**P**	**CD44p**	**P**	**HAS 1**	**P**	**HAS 2**	**P**	**HAS 3v1**	P
		**gene exp. values**		**gene exp. values**		**gene exp. values**		**gene exp. values**		**gene exp. values**	
	**∑= 120**	**median (range**)		**median (range**)		**median (range**)		**median (range**)		**median (range**)	
Age											
≤ 65	57	2.400 (0.00-13.43)	0.762	0.320 (0.00-1.92)	0.253	0.08 (0.0-7.55)	0.603	0.51 (0.01-7.96)	0.102	10.62 (0.069-50.82)	0.065
> 65	63	2.420 (0.06-14.76)		0.260 (0.00-1.24)		0.12 (0.0-103.04)		0.7 (0.0-13.68)		6.465 (0.0-35.65)	
Gender											
Male	93	2.340 (0.00-14.76)	0.494	0.305 (0.00-1.26)	0.185	0.07 (0.0-103.04)	0.072	0.52 (0.0-8.69)	0.059	7.73 (0.0-34.59)	0.608
Female	27	2.570 (0.29-13.40)		0.260 (0.00-1.92)		0.235 (0.0-7.55)		1.02 (0.01-13.68)		6.18 (0.12-50.82)	
Smoking											
yes	49	2.265 (0.06-13.40)	0.093	0.310 (0.02-1.65)	0.727	0.08 (0.0-7.55)	0.455	0.695 (0.0-13.68)	0.315	8.74 (0.04-35.65)	0.777
no	34	2.860 (0.67-14.76)		0.270 (0.00-1.92)		0.235 (0.0-103.04)		0.66 (0.02-8.69)		9.04 (0.02-50.82)	
Ta	31	1.460 (0.04-7.01)	**0.027**	0.300 (0.11-1.43)	0.2	0.02 (0.0-3.14)	0.066	0.125 (0.01-4.43)	**0.029**	11.43 (0.46-34.59)	0.392
T1	24	2.420 (0.19-10.12)	0.544	0.205 (0.06-1.20)	0.912	0.06 (0.0-0.99)	0.052	0.685 (0.0-13.68)	0.784	10.62 (0.0-30.13)	**0.021**
T2	21	2.890 (0.00-11.86)	0.96	0.170 (0.02-1.03)	0.215	0.21 (0.0-103.04)	0.137	0.68 (0.16-8.69)	0.797	4.98 (0.77-11.09)	0.613
T3	25	2.710 (0.66-9.18)	0.401	0.310 (0.00-1.92)	0.956	0.64 (0.01-7.23)	0.055	0.81 (0.01-7.96)	0.939	5.73 (0.02-50.82)	0.529
T4	19	3.815 (0.06-14.76)		0.470 (0.02-1.65)		0.125 (0.0-7.55)		1.14 (0.0-2.92)		3.26 (0.12-20.61)	
Non-inv.	55	1.685 (0.04-10.12)	**0.003**	0.270 (0.06-1.43)	0.744	0.02 (0.0-3.14)	**< 0.001**	0.22 (0.0-13.68)	**0.006**	7.675 (0.0-36.65)	**<0.001**
Invasive	65	2.875 (0.00-14.76)		0.310 (0.00-1.92)		0.26 (0.0-103.04)		0.805 (0.0-8.69)		1.98 (0.04-50.82)	
Grade											
G1	20	1.250 (0.16-3.89)	**0.021**	0.260 (0.16-1.20)	0.303	0.02 (0.0-3.14)	0.357	0.155 (0.02-13.68)	0.481	12.5 (0.28-29.38)	0.279
G2	43	2.445 (0.04-10.12)	0.179	0.250 (0.02-1.43)	0.224	0.03 (0.0-103.04)	**< 0.001**	0.36 (0.01-8.69)	**0.026**	8.875 (0.77-34.59)	**<0.001**
G3	57	2.810 (0.00-14.76)		0.330 (0.00-1.92)		0.27 (0.02-7.55)		0.83 (0.0-7.96)		3.925 (0.0-50.82)	
Low- grade	63	1.770 (0.04-10.12)	**0.006**	0.260 (0.28-0.46)	0.243	0.03 (0.0-103.04)	**< 0.001**	0.29 (0.01-13.68)	**0.014**	11.09 (0.28-34.59)	**<0.001**
High-grade	57	2.850 (0.00-14.76)		0.325 (0.00-1.92)		0.265 (0.0-7.55)		0.82 (0.0-7.96)		4.25 (0.0-50.82)	
Lymph node											
N0/Nx	97	2.240 (0.00-14.76)	**0.005**	0.270 (0.00-1.65)	0.514	0.07 (0.0-103.04)	**0.015**	0.61 (0.0-13.68)	0.302	7.675 (0.0-35.65)	**0.046**
N +	23	3.970 (0.06-11.86)		0.430 (0.00-1.92)		0.405 (0.0-5.62)		0.93 (0.11-7.96)		1.98 (0.04-5.82)	
Primary	74	2.225 (0.17-11.86)	**0.029**	0.305 (0.00-1.92)	0.957	0.125 (0.0-7.55)	0.575	0.645 (0.0-7.96)	0.464	8.21 (0.0-50.82)	0.902
Recurrent	46	2.760 (0.00-14.76)		0.310 (0.00-1.31)		0.115 (0.0-103.04)		0.55 (0.0-13.68)		6.95 (0.02-35.65)	

mRNA gene expression levels of HA-associated genes (RHAMM, CD44, HAS1, - 2 and ‑ 3v1) in tumor samples of 120 patients with BC disease. Characterization is stratified according to (A) age, gender and smoking status and (B) tumor stage, tumor grading, lymph node status and primary tumors versus recurrent tumor disease. Data were analyzed using univariate Cox proportional hazard survival regression analysis, total n=120.

### mRNA Expression

**Figure 1:**
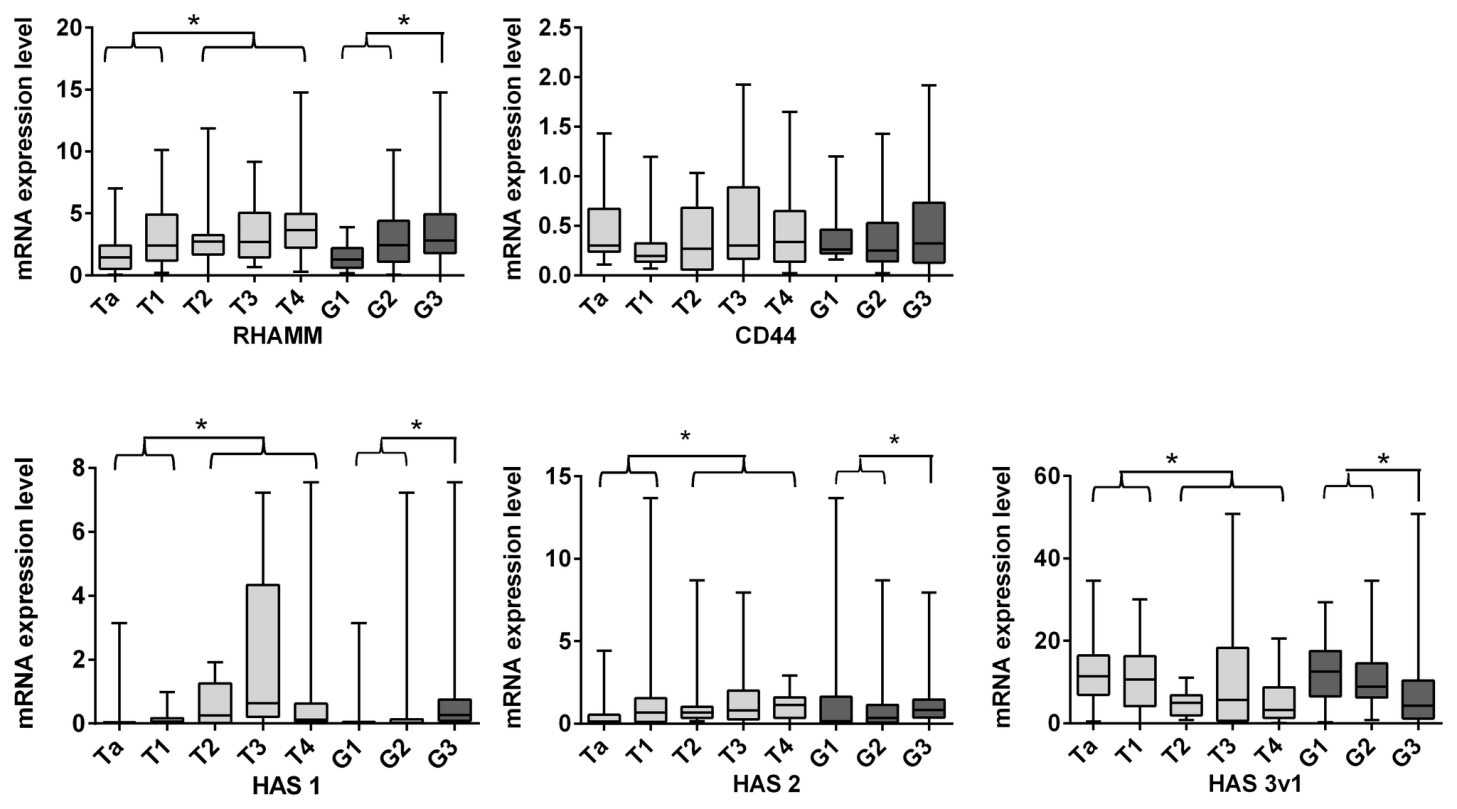
RHAMM mRNA expression is elevated in muscle-invasive, high grade tumor stages compared to non-invasive low grade tumor stages. RHAMM, CD44, HAS1, HAS2 and HAS3v1 mRNA expression in human BC tissue, shown as boxplot-diagrams in 25% to 75% intervals with minimum to maximum whiskers. Real-time-RT-PCR (total patients n=120, non-invasive tumors n=55, invasive tumors n=65; Ta n=31, T1 n=22, T2 n=21, T3 n=25, T4 n=19; low grade n=63, high grade n=57).

To assess their association with tumor infiltration and dedifferentiation, HAS1, HAS2, HAS3v1, RHAMM and CD44 mRNA expression levels were determined in BC tissue of 120 patients as fold of control tissue, using quantitative real-time-RT-PCR (Table 1, Figure 1). As a result RHAMM expression levels were elevated in infiltrating tumors compared to non-invasive tumors (RHAMM: non-invasive tumors 1.69 fold (Ta + T1), invasive tumors 2.88 fold (T2-4), p=0.003). Furthermore, mRNA expression of HAS1 and HAS2 were significantly upregulated in muscle-invasive tumor stages (pT2, pT3 and pT4). In contrast, HAS3v1 was elevated in all tumor stages compared to healthy bladder tissue but decreased with increased tumor staging (non-invasive tumors 11.26 fold (Ta + T1) and invasive tumors 4.72 fold (T2-4), p<0.001; data calculated as median). Furthermore, CD44 was not upregulated compared to controls and not regulated with respect to muscle-invasiveness.

Similar results were obtained with respect to tumor cell grading. RHAMM, HAS1 and HAS2 mRNA expression levels were significantly increased in higher tumor gradings (G1 versus G2-3; RHAMM, 1.77 fold in low grade, 2.81 fold in high grade tumors, p=0.006; HAS1, 0.02 fold in low grade and 0.26 fold in high grade specimens, p<0.001; HAS2, 0.29 fold in low grade and 0.82 fold in high grade tumors, p=0.014). Again HAS3v1 mRNA expression levels were strongly upregulated in G1 compared to control but decreased with higher tumor grading (HAS3, 10.91 fold of control in low grade versus 3.93 fold in high grade findings, p<0.001). In addition, CD44 showed no differences also with respect to tumor cell grading.

Lymph node status was also associated with significant differences in expression levels of several genes. Tumor tissue of lymph node positive patients showed higher RHAMM and HAS1 mRNA expression levels compared to those with negative lymph node status. HAS3v1 expression levels were decreased in patients with positive lymph node status (Table 1 B) compared to lymph node negative specimens. Interestingly, RHAMM mRNA expression was the only HA-associated gene that was significantly increased in recurrent tumors compared to primary tumors.

### Protein Expression in human Tumor Samples

**Figure 2:**
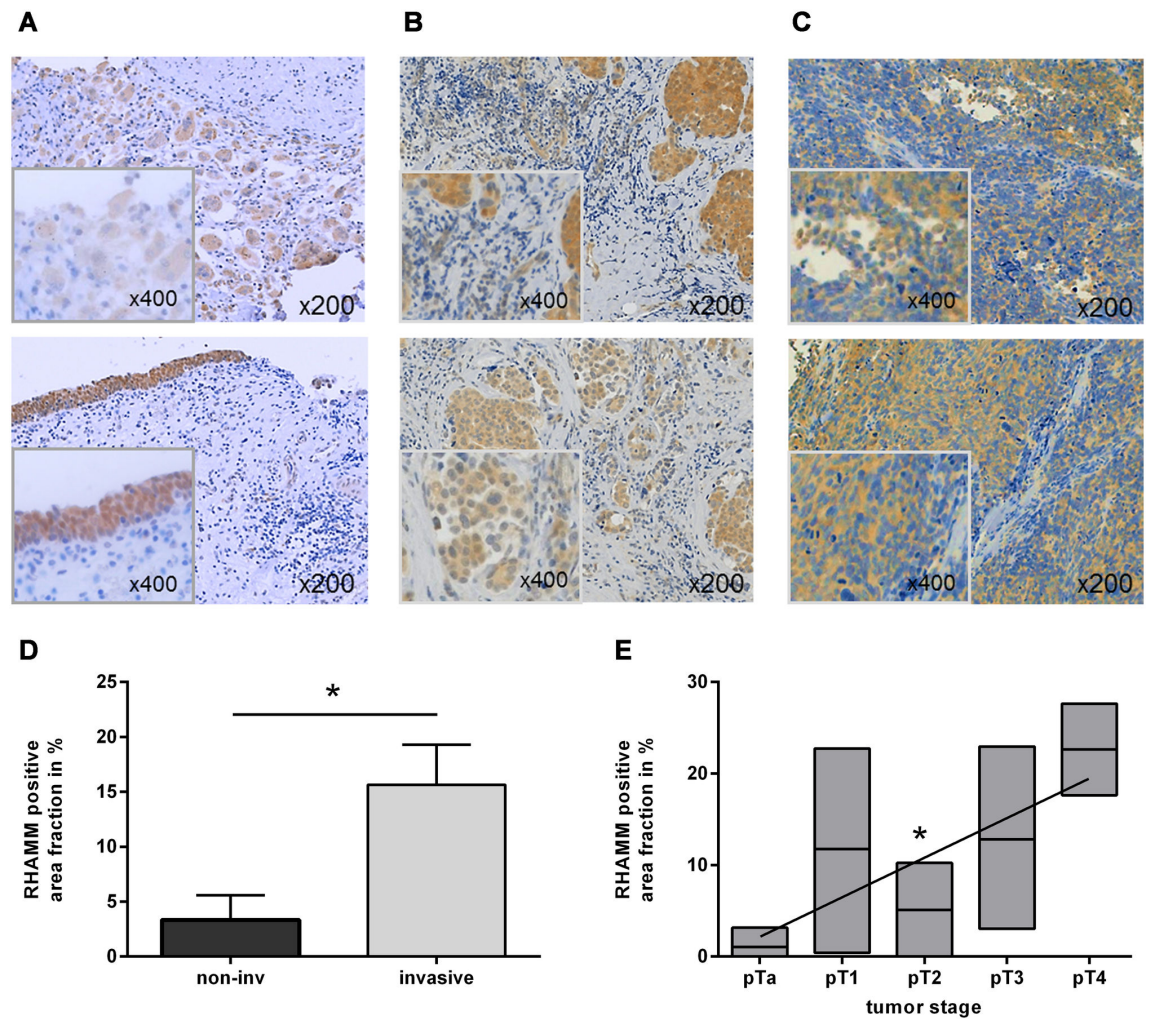
Increased RHAMM staining during tumor progression. (**A**-**C**) representative immunohistological staining of RHAMM in paraffin-embedded human BC tissue. (**A**) in nonmuscle-invasive tumor stages T1 the bladder structure is still preserved. (B) in tissue of high stage tumors the malignant cells start to proliferate and form independent cell clusters surrounded by stromal cells (pT2). (**C**) in non-organ confined tumor stages (pT4), the bladder structure is severely disturbed by infiltrating tumor cells. (**D**) the area fraction of RHAMM positive cells is elevated in highly muscle-invasive tumor stages as determined by image analysis (non-invasive, n=8; invasive n=12; *p=0.03, t-test). (**E**) correlation of RHAMM positive area fraction with higher tumor stages (n=4; *p=0.0129, Pearson-correlation); mean ± SEM.

To further characterize the expression of RHAMM in human BC immunohistochemical staining was performed in 20 representative cases (4 each in pTa-pT4). RHAMM staining showed rarely stained cells in papillary non-invasive tumors (Figure 2 A). Normal bladder tissue morphology was not affected in these papillary superficial tumors and RHAMM was predominantly located in the urothelium. With emerging muscle invasion, RHAMM positive stained tumor cells start to form isolated cell clusters within the muscular tissue of the bladder wall (Figure 2 B) and overall staining intensity and tissue content of RHAMM positive cells increased. In dedifferentiated deep invasive and non-organ confined tumors in histopathological stage pT4, RHAMM positive tumor cells are ubiquitously distributed in human tumor samples. Predominantly tumor cells, but also to a lesser extend stromal cells expressed RHAMM (Figure 2 C). RHAMM positive area fraction was 3.3% in non-invasive tumors (pTa-1) compared to 21.6% in muscle-invasive findings (pT2-4, p=0.03, Figure 2 D). The increase of RHAMM also correlated with increasing tumor stage (p=0.0129, Figure 2 E).

### Correlation between Disease-specific Survival and Gene Expression

Patients with high and low expression of the respective genes were divided into two groups using the median value as cut-off. Results of univariate cox analysis are listed in Table 2. Age, sex and smoking status showed no impact on survival rates. As expected, high tumor stage, high grade and positive lymph node status showed significant negative correlation with disease-specific and overall survival. Of note, high RHAMM and HAS2 mRNA expression levels are significantly associated with poor disease-specific and overall survival. In contrast, high HAS3v1 mRNA expression correlated with favorable overall survival and cancer-specific survival.

**Table 2:** In patients with muscle-invasive tumors poor disease-specific survival is associated with high RHAMM and high HAS2 mRNA expression levels.

Table **2A** **:**									
**Variables**		**Overall survival**					**Disease-specific survival**		
	**n**	**HR**		**95% CI**		**P**	**HR**	**95% CI**	**P**
Age									
≤ 65	57	ref.					ref.		
> 65	63	1.111		0.715-1.726		0.64	1.171	0.701-0.959	0.546
Sex									
Female	27	ref.					ref.		
Male	93	0.677		0.411-1.115		0.126	0.643	0.362-1.142	0.132
Smoking									
no	49	ref.					ref.		
yes	34	0.897		0.535-1.505		0.681	0.604	0.322-1.100	0.099
Table 2B:									
Stage									
Non-inv. (Ta-T1)	55	ref.					ref.		
Invasive (T2-T4)	65	3.416		2.110-5.529		<0.001	5.766	3.039-10.940	<0.001
Grade									
Low-grade	63	ref.					ref.		
High-grade	57	3.124		1.964-4.969		<0.001	3.834	2.189-6.713	<0.001
Lymph node status									
N- / Nx	97	ref.					ref.		
N+	23	3.706		2.198-6.250		<0.001	5.066	2.871-8.938	<0.001
Prior Recurrence									
Primer	74	ref.					ref.		
Recurrent	46	1.341		0.848-2.120		0.21	1.206	0.705-2.065	0.494
Table 2C:									
RHAMM									
low	56	ref.					ref		
high	56	2.499		1.556-4.013		<0.001	. 3.355	1.887-5.965	**<0.001**
CD44p								
low	55	ref.					ref.		
high	55	0.848		0.533-1.347		0.848	0.877	0.511-1.504	0.633
HAS 1								
low	49	ref.					ref.		
high	46	1.626		0.983-2.688		0.058	1.731	0.970-3.089	0.063
HAS 2								
low	53	ref.					ref.		
high	53	1.74		1.080-2.803		0.023	1.794	1.033-3.118	0.038
HAS 3v1								
low	55	ref.					ref.		
high	54	0.565		0.352-0.906		0.018	0.586	0.340-1.011	0.055

Overall and disease-specific survival in patients with urothelial TCC stratified according to (A) patient characteristics: age, gender and smoking status (B) disease-specific clinical parameters: tumor stage, tumor grading, lymph node status and primary tumors versus recurrent tumor disease (C) HA-associated genes (RHAMM, CD44, HAS1, - 2 and - 3v1). Univariate Cox proportional hazard survival regression analysis was used for data analysis, total n=120. Abbreviations: HR - hazard ratio, CI – confidence interval, ref. – referent.

**Figure 3:**
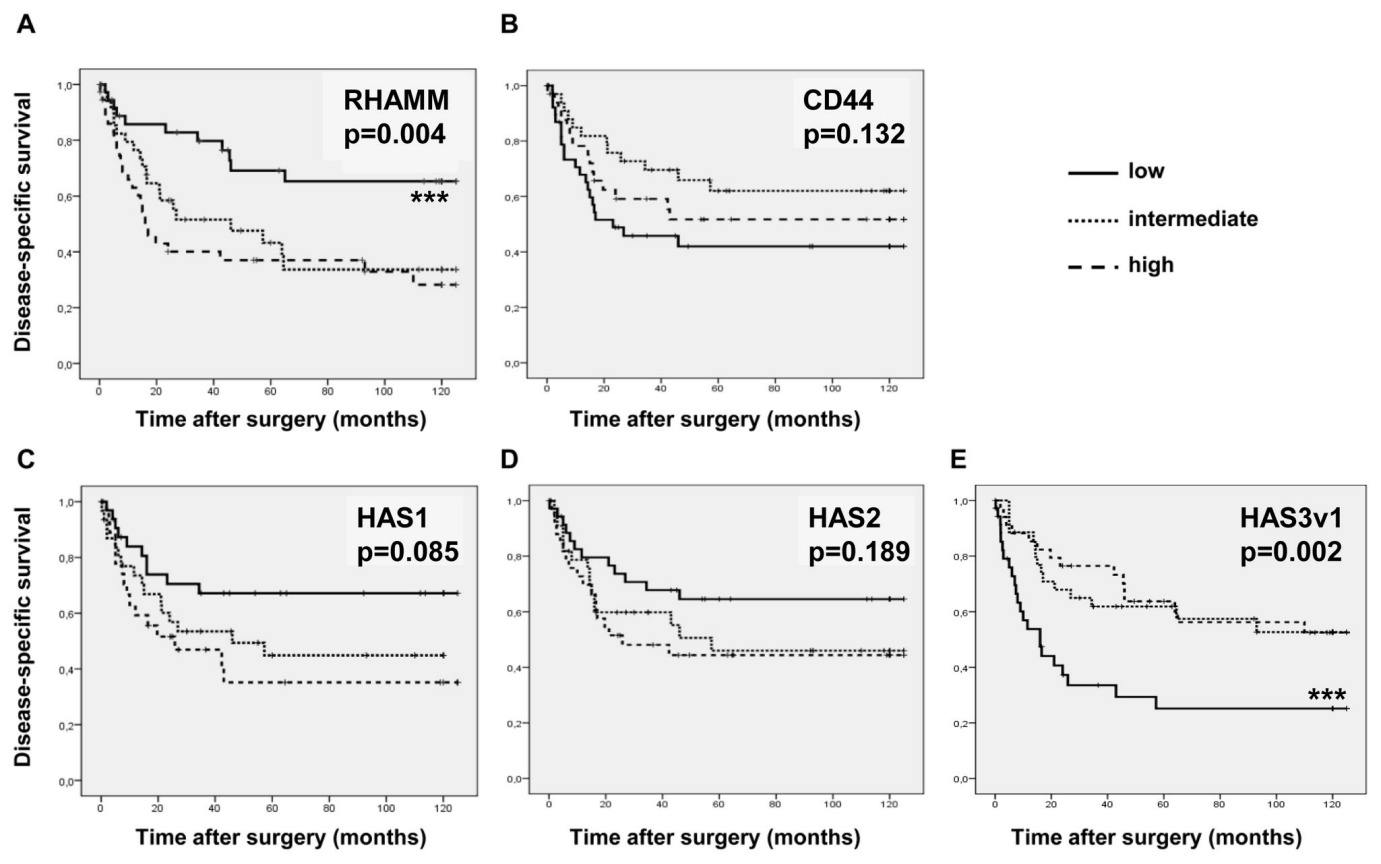
High and intermediate RHAMM as well as low HAS3v1 mRNA expression levels are associated with reduced cancer-specific survival. The patient cohort was split into three subgroups consisting of high, intermediate and low expression of either RHAMM, CD44, HAS1, -2 or -3 mRNA (**A**-**E**). Kaplan-Meier survival probability test was performed showing significant increase in the risk of cancer-related death for the subgroups of high and intermediate RHAMM mRNA expression levels compared to low RHAMM mRNA expression (**A**). Transcript levels of CD44 (**B**), HAS1 (**C**) and HAS2 (**D**) were associated with significant changes of disease-specific survival. Notably, high HAS3v1 mRNA expression was significantly associated with lower risk of cancer-specific death compared to the subgroups with low and intermediate HAS3v1 mRNA expression (**E**). Data from a total of n=120 patients; analysis was performed in three groups (n=40 each) of high, intermediate and low mRNA expression of the respective genes.

For Kaplan-Meier survival analysis the patient population was divided into three groups, consisting of 40 patients each. Notably significantly increased cancer-related mortality was associated with intermediate and high RHAMM mRNA expression (Figure 3A). Expression of low HAS3v1 mRNA levels also was associated with poor cancer-specific survival (Figure 3E). In contrast, high levels of HAS-1, and HAS-2 expression showed only a trend to decreased survival and CD44 was not associated with differences in the disease-specific survival curves (Figure 3B-D). To assess whether the analyzed gene expression levels provide independent prognostic information, cox multivariate hazard regression models were used. Parameters, influencing survival in univariate analyses, were included in the multivariate models. Importantly, high RHAMM mRNA expression levels and lymph node positivity were found to be independently associated with increased cancer-specific mortality risk (Table 3, HR=2.179, 95% CI 1.182-4.017, p=0.013, and HR=2.389, 95% CI 1.227-4.651, p=0.010).

**Table 3:** High RHAMM mRNA expression levels is an independent prognostic marker for disease-specific survival in patients with BC.

	**Disease-specific survival**			**Metastasis-free survival**		
**Variables**	**HR**	**95% CI**	**p-value**	**HR**	**95% CI**	**p-value**
Stage (invasive – non-invasive)	2.165	0.922-5.087	0.076	6.228	1.628-24.281	**0.008**
Grade (low grade-high grade)	1.960	0.923-4.165	0.080	0.639	0.188-2.170	0.473
Lymphnodes (N+ -N0)	2.389	1.227-4.651	**0.010**	-	-	-
RHAMM (low - high)	2.179	1.182-4.017	**0.013**	1.732	0.663-4.529	0.262
HAS 1 (low - high)	0.584	0.311-1.097	0.094	0.502	0.149-1.695	0.267
HAS 2 (low - high)	1.391	0.791-2.448	0.252	0.126	0.480-3.306	0.640
HAS 3v1 (low - high)	0.905	0.510-1.604	0.731	1.087	0.370-3.197	0.879
CD44p (low - high)	0.635	0.363-1.110	0.111	0.649	0.244-1.726	0.386

Data analysis calculating the influence of mRNA expression levels on patient survival, independent from the patient-specific parameters mentioned in Table 2. High RHAMM mRNA expression in tumor tissue was found to be an independent (p=0.013) prognostic marker for disease-specific survival (multivariate cox proportional hazard survival regression analysis, n=120). Abbreviations: HR - hazard ratio, CI – confidence interval.

### Lentiviral RHAMM knock-down inhibits Xenograft Tumor Growth in vivo

**Figure 4:**
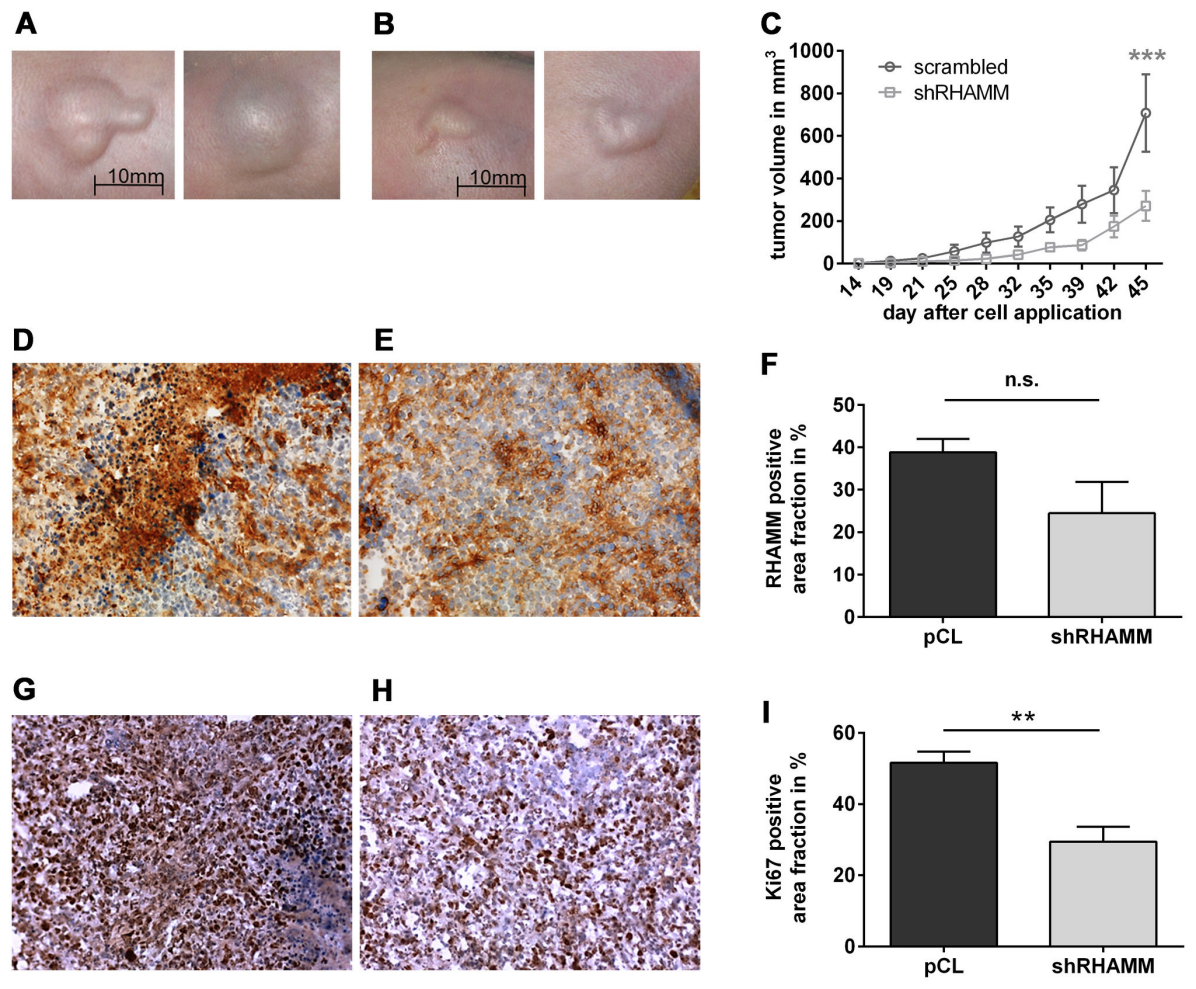
RHAMM knock-down reduces tumor growth in vivo. (**A**-**C**) xenograft tumor growth in athymic mice is reduced when human J82 BC cells were transduced with shRHAMM prior to injection. (**A**) control group that received J82 cells transduced with scrambled shRNA; (**B**), tumors derived from injection of J82 cells transduced with shRHAMM. (**C**) 45 days after cell application tumor volume was significantly reduced by shRHAMM. (**D**-**F**) immunostaining of RHAMM and Ki67 (**G**-**I**). Data represent mean ± SEM, n=7 for scrambled and shRHAMM each, *p < 0.05).

To address experimentally the possible role of RHAMM in bladder tumor growth, a xenograft model using J82 cells was established in nude mice. J82 cells transduced with either scrambled shRNA as control and shRHAMM were subcutaneously injected and tumor growth monitored. As a result 45 days after injection of tumor cells reduced tumor size was found in mice that received J82 cells with knock-down of RHAMM compared to the control group (450 mm^3^ ± 188.2 mm^3^ for shRHAMM and 711.5 mm^3^ ± 201.2 mm^3^ for control, p<0.001, Figure 4 A-C). Histological analysis revealed that tumors were capsuled and confined to the site of cell injection. Metastatic growth was not seen in any of the animals, neither in shRHAMM group nor in controls. At the end of the experimental period some of the tumors in the control group showed necrosis likely due to insufficient vascular supply in the center of the solid tumor tissue. RHAMM immunostaining showed even after 47 days still a trend towards reduced expression in the shRHAMM group (24.5% area fraction in shRHAMM versus 38.8% in control tumors; p=0.0984, Figure 4 D–F). To elucidate the underlying mechanisms of reduced xenograft tumor growth the proliferative activity was assessed by Ki67 staining. Of note, proliferation of tumor cells in tumors derived from RHAMM knock-down cells was significantly decreased compared to control (Figure 4 G-I) suggesting that reduced proliferation was responsible for the inhibition of tumor growth. In line with this conclusion the staining pattern of Ki67 revealed that proliferation occurred predominantly in tumor cells whereas stromal fibroblasts or inflammatory cells were mostly negative for Ki67.

### In vitro Analysis of Bladder Carcinoma Cell Phenotype

**Figure 5:**
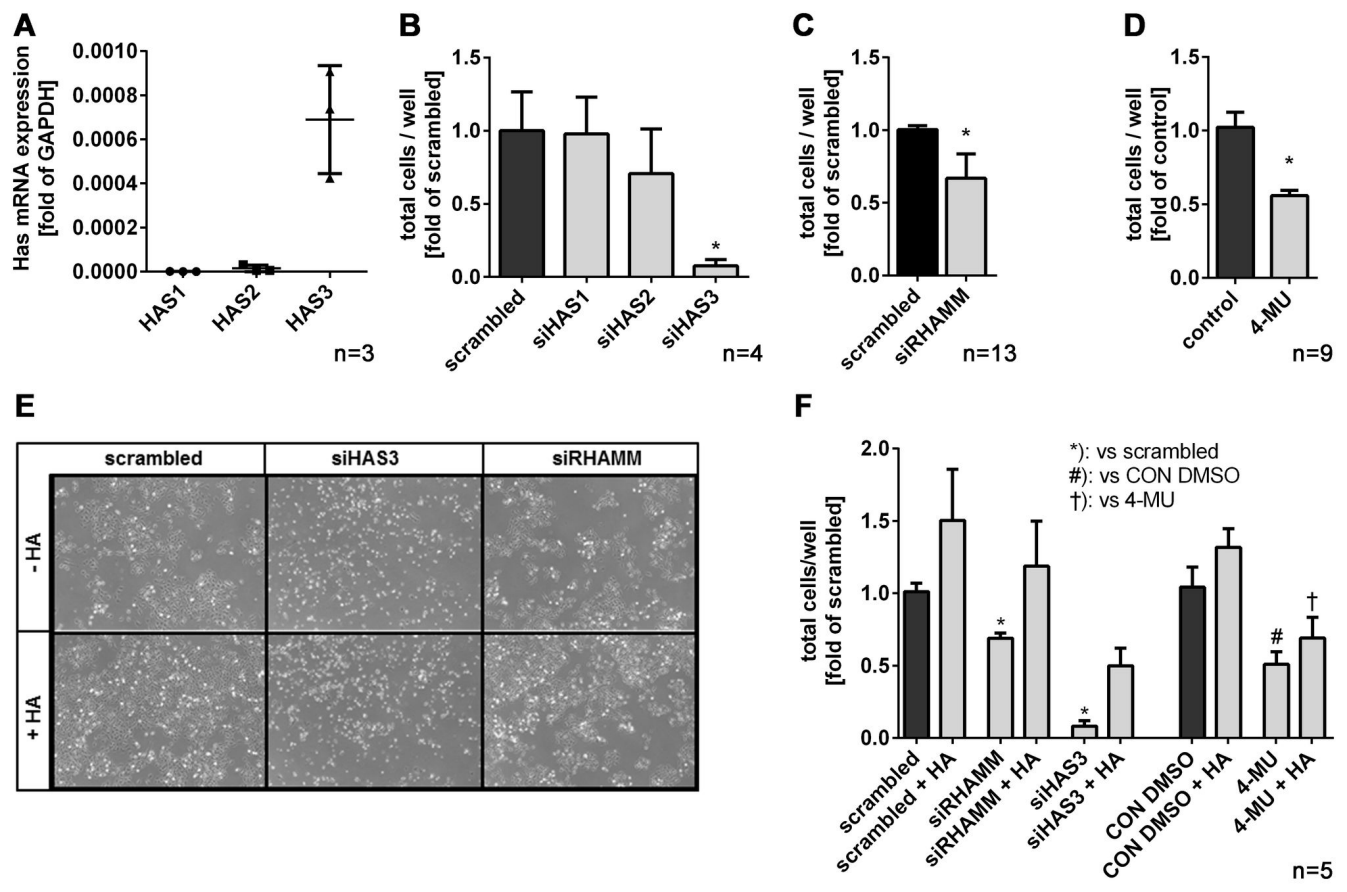
Inhibition of HA synthesis and RHAMM knock-down reduce cell proliferation in vitro. (**A**) HAS1-, HAS2- and HAS3 mRNA expression in J82 BC cells as determined by RT-PCR. (**B**-**D**) proliferation of J82 cells 4 days after transfection with either HAS1-, HAS2-, HAS3- and RHAMM siRNA in 10% FCS or after 2 days of treatment with 4-MU (300 µM). (**E**) representative bright field images of J82 cells 4 days after knock-down of HAS3 and RHAMM plusminus 100 µg HA/ml (Healon 5). (**F**) proliferation expressed as J82 cells/well treated either with siRNA against HAS3 or RHAMM or 4-MU for 2 days and the respective controls. HA (100 µg/ml) was added to test whether the effects could be rescued by exogenous HA; mean ± SEM, *, p<0.05.

J82 cells were analyzed with respect to HAS isoenzyme expression by RT-PCR which revealed that HAS1 and HAS2 were expressed at very low levels and that HAS3 was the main isoenzyme (Figure 5A). Four days after knock-down of HAS1 and HAS2 no effects on proliferation were detected as judged by cell counting (Figure 5B). In contrast, knocking down HAS3 caused a strong decrease in cell number and even a loss of cells as evident from the microscopic images shown in Figure 5E. Furthermore, lentiviral knock-down of RHAMM by shRNA as used in the animal experiment inhibited proliferation both in medium containing 10% serum Figureand in serum free medium (not shown). For further mechanistic studies RHAMM was knocked down with siRNA resulting also in reduced cell proliferation (Figure 5C). Inhibition of HA synthesis with 4-MU repressed J82 proliferation as well (Figure 5D). Subsequently, exogenous HA was added to the respective controls and cells treated with siHAS3, siRHAMM and 4-MU. In line with a pro-proliferative role of HA, the anti-proliferative effects of siHAS3 and 4-MU were partially reverted and controls were stimulated by HA (Figure 5F). Interestingly, however, the anti-proliferative effect of siRHAMM was rescued by exogenous HA as well (Figure 5F), which is suggestive of RHAMM-independent pro-proliferative HA signaling in J82 BC cells.

**Figure 6:**
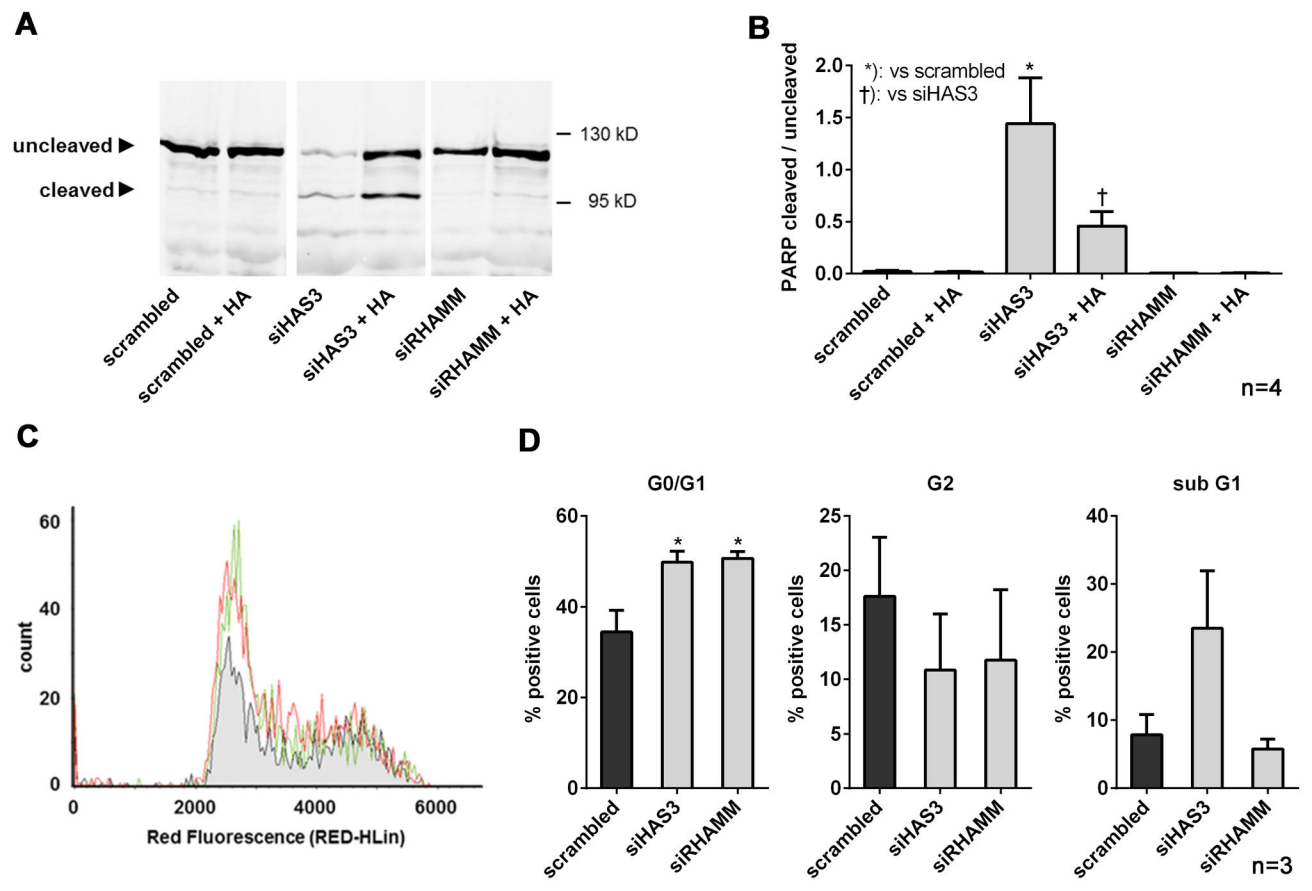
Knock-down of HAS3 causes apoptosis in bladder carcinoma cells *in vitro*. Analysis of apoptosis and cell cycle progression were performed 4 days after transfection with siRNA targeting HAS3 and RHAMM as detailed also in Figure 5. (**A**) immunoblotting of PARP and cleaved PARP after knock-down of HAS3 and RHAMM by siRNA plusminus 100 µg/ml HA. (**B**) apoptotic activity was expressed as the ratio of cleaved and uncleaved PARP; experiments were performed as described in A. (**C**), cell cycle analysis by FACS; black line, scrambled; green line, siRHAMM; red line; siHAS3; representative FACS histograms. (**D**) quantitative analysis of FACS analysis with respect to G0/G1 phase representing cell cycle arrest; G2 phase representing premitotic cells and sub G1 indicating apoptotic cells; mean ± SEM, *, p<0.05.

In addition, it was addressed whether knock-down of HAS3 and RHAMM affected apoptosis in J82 cells. siRHAMM did not induce apoptosis as evidenced by PARP cleavage (Figure 6A,B) and the percentage of cells in sub G1 phase (Figure 6C,D). In contrast, knocking down the main HAS isoenzyme (HAS3) caused strong cleavage of PARP in J82 cells which was rescued by application of exogenous HA (Figure 6A,B). The pro-apoptotic effect of siHAS3 was corroborated by an increase of the cells in sub G1 as evidenced by FACS analysis (Figure 6D). In addition, the anti-proliferative effects of siRHAMM and siHAS3 were also confirmed by increased percentage of cells in G0/G1 phase (Figure 6D).

**Figure 7:**
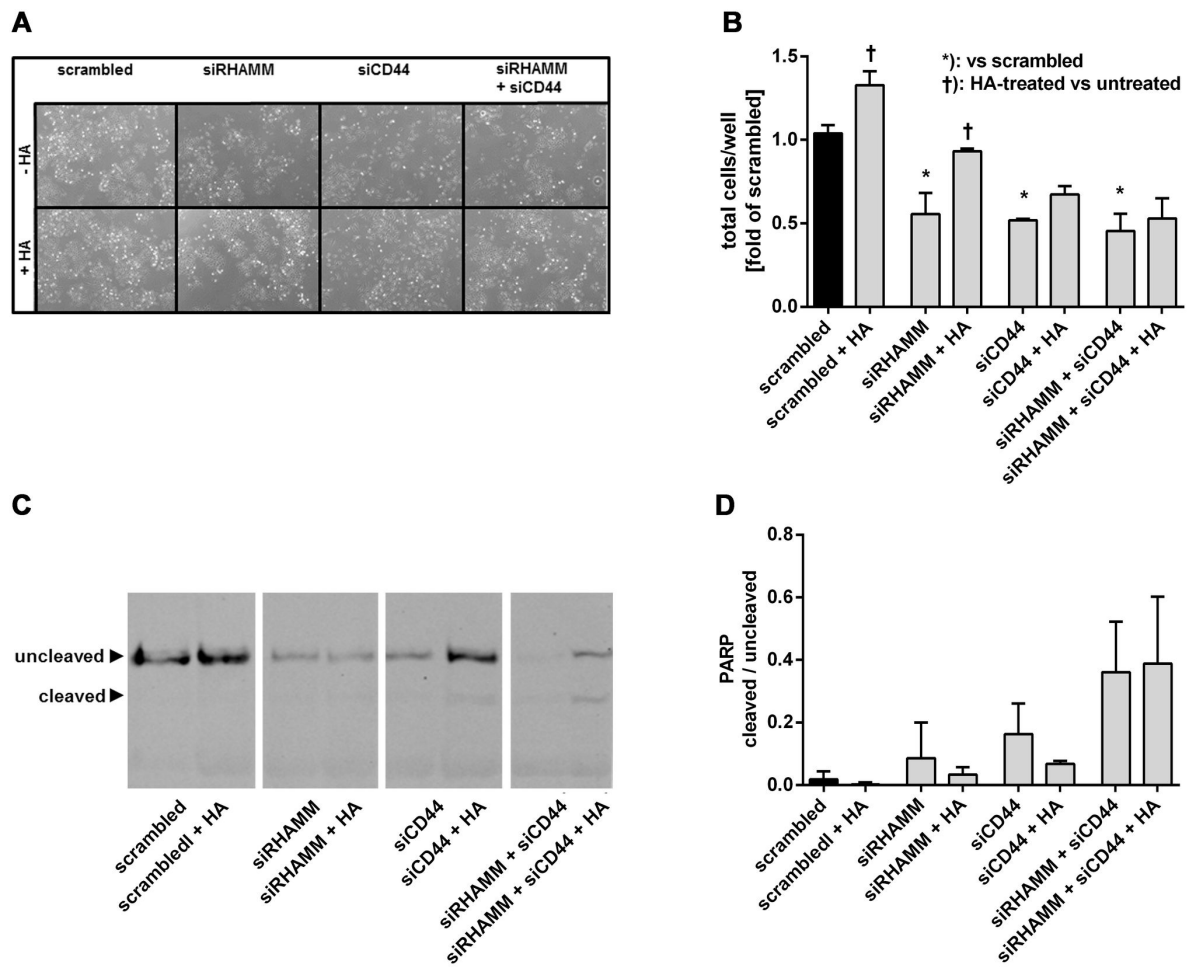
Knock-down of CD44 inhibits proliferation of bladder carcinoma cells *in vitro*. (**A**) representative bright field images of J82 cells 4 days after knock-down of RHAMM, CD44 and RHAMM plus CD44 plusminus 100 µg HA/ml (Healon 5) in 10% FCS. (**B**) proliferation of J82 cells after knock-down of RHAMM, CD44 and CD44 plus RHAMM in J82 cells 4 days after transfection plusminus exogenous HA (100 µg/ml). (**C**) analysis of apoptosis was performed 4 days after transfection with siRNA as in A and B by immunoblotting of PARP and cleaved PARP. (**D**) PARP cleavage was quantified as the ratio of cleaved and uncleaved PARP; mean ± SEM, *, p<0.05.

The data presented in Figure 5F revealed that the anti-proliferative effect of RHAMM was rescued by addition of exogenous HA suggestive of RHAMM independent pro-proliferative HA signaling in J82 cells. Therefore, siCD44 was used alone and in combination with siRHAMM to analyze whether J82 cells employ CD44 generally or in the absence of RHAMM to execute pro-proliferative HA signaling. These experiments revealed that knock-down of CD44 indeed inhibited proliferation (Figure 7A,B) and that this effect was not rescued by HA, which is in line with pro-proliferative HA/CD44 signalling in J82 cells. When CD44 was knocked down in addition to RHAMM a strong anti-proliferative effect was detected that was not rescued by HA (Figure 7A,B). Furthermore, increased apoptosis was evident if both CD44 and RHAMM were knocked down (Figure 7C,D). All together these *in vitro* experiments suggest that RHAMM promotes J82 proliferation at least in part independently from HA and that CD44 can compensate for RHAMM down-regulation through HA-dependent pro-proliferative signaling.

## Discussion

Patients suffering from urothelial transitional cell cancer of the bclinical outcometheir prognosis. Patients with non-muscle-invasive BC (pTa and pT1 low grade) are treated by TUR of the tumor. In most cases this procedure combines diagnostic and curative treatment leading to excellent tumor control. Within TUR representative tissue samples can be taken for histopathological diagnosis and determine the necessity of more aggressive treatment. For many patients this local procedure can become a long-term bladder preserving strategy. However, 3-5% of the patients suffering from non-muscle-invasive BC experience progression to muscle-invasive disease and need radical treatment. Only about 50% of the patients with muscle-invasive BC benefit from the radical operation with long-term disease-free survival [36,37]. To develop more individualized therapeutic strategies, it is crucial to identify tumor-related parameters allowing risk stratification within the group of muscle-infiltrating transitional cell carcinomas and in the long run represent potential therapeutic targets.

In this regard HA and HA-associated genes, such as HAS isoenzymes, HA receptors and HA catabolic enzymes, appear to be interesting because these genes are of pathophysiological significance and of diagnostic value in various cancer entities [38]. Interestingly, the association of HA with cancer parenchyma or with tumor stroma is specific for certain cancer types and can also be stage-dependent [6]. These observations suggest that HA affects both tumor cells and stromal cells. Furthermore, it is likely that tumor-associated fibroblasts can secrete HA that is subsequently utilized by tumor cells [39]. The expression of HA-related genes has been studied also in BC. As a result, especially HAS1, Hyal1 and urinary HA excretion were found to be associated with BC progression and/or prognosis [18,22,25,29,37,40,41].

The present investigation revealed a stage-dependent increase of RHAMM expression in human BC specimens as evidenced by real-time RT PCR and by immunohistochemistry. Of note, high RHAMM expression levels were associated with increased disease-specific and increased overall mortality. Importantly, in this study we present for the first time that RHAMM mRNA expression is even an independent risk factor in muscle-invasive BC. Subsequently knock-down of RHAMM caused significant inhibition of xenograft tumor growth in nu/nu athymic mice. This reduction of tumor progression was likely caused by inhibited proliferation of BC tumor cells as evidenced by Ki67 staining. Further *in vitro* experiments verified the antiproliferative effect of RHAMM knock-down.

In addition, the *in vitro* experiments revealed that J82 cells react with decreased proliferation to inhibition of HA synthesis achieved either by knock-down of the main HAS isoenzyme, HAS3, or by application of 4-MU. Thus emphazising the importance of HA for BC cell proliferation. Interestingly, growth inhibition in response to siRHAMM was overcome by addition of exogenous HA. These results could indicate flexibility of J82 cells to execute HA signaling through CD44 if the RHAMM pathway is blocked. This hypothesis was supported by the finding that the anti-proliferative effect of siRNA targeting CD44 was not rescued by HA in BC cells. Similarly the simultaneous knock-down of both CD44 and RHAMM caused inhibition of proliferation that was not sensitive to HA anymore. The data, however, also suggest that the anti-proliferative effect of siRHAMM was not based on the abrogation of endogenous HA-signaling through RHAMM. Instead it must be considered that RHAMM may promote J82 proliferation independent of HA-mediated signaling, because it is known that intracellular RHAMM activates kinase signaling and that RHAMM is associated with microtubules during mitosis [17]. Both of these intracellular RHAMM actions could contribute to the pro-proliferative function of RHAMM in TCC independently of HA. RHAMM has been associated with increased phenotypic activation of various tumor cells. For example RHAMM is involved in cell motility, proliferation and migration of prostate cancer, breast cancer, esophageal cancer and lymphoma [15,39,42,43]. High RHAMM mRNA expression levels were also associated with unfavourable outcome of patients with colorectal cancer and B-cell lymphoma [43,44]. These findings have fostered the idea to use RHAMM as a target for therapy in acute myeloid leukemia and multiple myeloma and this is now being evaluated by vaccination against RHAMM in clinical trials [45].

Previous studies revealed that increased HAS1 isoenzyme [22,23] and Hyal1 expression in human BC were predictive for muscle-invasive tumor growth, metastasis and poor disease-specific survival [25,29]. Our data, in line with previous reports, showed increased HAS1 expression to be associated with invasive tumor growth and high malignity in BC.In this regard HAS1 was significantly upregulated in muscle-invasive tumors and in dedifferentiated G3 high grade compared to G1-2 low grade BC. High expression levels of HAS1 were associated with a trend towards poor disease-specific survival. Furthermore, HAS2 was upregulated in high grade compared to low grade carcinomas and tended to be associated with worse overall and disease-specific survival. These data are consistent with the findings of several *in vitro* studies showing the enhancing impact of HAS2 overexpression on tumor growth and tumorigenicity in human prostate and BC cell lines [46,47]. However, in contrast to RHAMM the prognostic value of HAS2 was not independent from other strong prognostic factors such as lymph node invasion, histological grading and tumor stage. This is supported by the recent finding that HAS2 expression by itself is not able to predict BC outcome [25]. HAS3 showed yet another expression pattern. HAS3 was initially strongly upregulated in Ta and T1 and in G1 tumors and declined during progression. Further analysis showed that high HAS3v1 mRNA levels were associated with lower mortality. In accordance, overexpression was shown to inhibit tumor growth in an orthotopic mouse model with prostate cancer cell lines [45]. Mechanistically, this might be explained by reduced tumor cell adhesion, angiogenesis and tumor progression as previously described for HAS3 overexpression [47]. However, in other cancer cells, e.g. oesophageal squamous cell carcinoma cells HAS3, promotes tumor growth as shown by HAS3 knock-down or application of the HAS-inhibitor 4-MU [14,39]. Therefore, another possible explanation for the association of low HAS3 expression with a more favorable outcome may be that HAS3 is elevated in early tumor stages and HA synthesis is taken over by HAS1 and HAS2 during progression. That way high HAS3 expression would be indicative for yet less progressed tumors. The assumption that HAS3 is indeed important for TCC progression was supported by the present *in vitro* results revealing dramatic inhibition of J82 cell proliferation and induction of apoptosis in response to HAS3 knock-down.

Strong evidence now shows that the HA system is implicated in BC progression and that it can have also predictive value with respect to mortality. However, as indicated above differences regarding the relative importance of the different HA- associated genes and the expression levels exist between the studies including the present study. These discrepancies might be explained by differences in the characteristics of the patient cohorts and controls. The strength of the present study was that (i) the numbers in the two groups (55x non-invasive, 65x muscle-invasive disease) were similar, (ii) patients did neither receive neoadjuvant chemotherapy or BCG-immunotherapy (Bacillus Calmette-Guérin) and (iii) the control tissue was derived from patients that did not suffer from urothelial carcinoma.

In light of the literature and the present findings it is likely that HAS1 but also HAS2 and HAS3 contribute to HA synthesis and TCC progression. Of note, only RHAMM may serve as an independent marker of poor prognosis in muscle-invasive BC. However, it cannot be concluded whether RHAMM serves bladder carcinoma cells as a receptor of endogenous HA or whether RHAMM promotes bladder carcinoma progression independent of extracellular HA.

Prognostic markers may help to improve the accuracy of risk stratification of cancer patients and therefore may provide important data to optimize therapeutic decisions. The long-term postoperative treatment of other urological tumors like prostate cancer or testicular cancer is unimaginable without tumor markers during follow-up. In prostate cancer, prostate-specific antigen, in testicular cancer human choriogonadotropin and alpha-fetoprotein play important roles in the daily decision making. Clinical data then showed that analysis of tumor tissue itself immediately after resection reveals individual risk factors that can be used to optimize the anti-tumor strategy from active-surveillance to immediate chemotherapy. This approach is already practiced in e.g. testicular cancer [47,48]. To date, no prognostic markers are available for risk stratification of BC patients. In this study we present for the first time RHAMM mRNA expression as an independent prognostic factor in BC. Furthermore, we show the causal involvement of RHAMM in BC proliferation *in vitro* and *in vivo*in tumor growth.
